# Pacific Gulls (*Larus pacificus*) as Potential Vectors of *Coxiella burnetii* in an Australian Fur Seal Breeding Colony

**DOI:** 10.3390/pathogens12010122

**Published:** 2023-01-11

**Authors:** Brett R. Gardner, Jasmin Hufschmid, John Stenos, Mythili Tadepalli, Grace Sutton, Aymeric Fromant, Yonina Eizenberg, Johanna J. Geeson, John P. Y. Arnould

**Affiliations:** 1 Melbourne Veterinary School, The University of Melbourne, Parkville, VIC 3030, Australia; 2 Australian Rickettsial Reference Laboratory, University Hospital Geelong, Bellerine Street, Geelong, VIC 3220, Australia; 3 School of Life and Environmental Sciences, Deakin University, Geelong, VIC 3125, Australia; 4 Centre d’Etudes Biologiques de Chizé (CEBC), UMR 7372 CNRS-La Rochelle Université, 79360 Villiers-en-Bois, France

**Keywords:** oral cloacal swabs, sea bird, southeastern Australia, abortion, placenta, marine mammal gestational loss, zoonoses

## Abstract

Recently, *Coxiella burnetii* has been described as a novel pathogen potentially contributing to decreased pup production in Australian fur seals (AusFS, *Arctocephalus pusillus doriferus*). Pacific gulls (PGs, *Larus pacificus*) are known to scavenge AusFS placental material during the fur seal breeding season. It is hypothesized that PGs may act as vectors for this pathogen. In the present study, cloacal swabs, oral swabs and serum were collected from PGs on Kanowna Island (KI, an AusFS breeding colony) and a nearby island, Seal Island (SI), not occupied by pinnipeds. All sample sets were evaluated with qPCR for the com1, htpAB and IS1111 markers. Most oral and cloacal swabs from KI tested positive on both the com1 (94.1%; 88.2%) and htpAB targets (76.5%; 76.5%). Amplification was very low from the SI oral swabs and cloacal swabs. Only the KI serum samples had amplification (17.7% for both com1 and htpAB). There was no IS1111 amplification in either colony. The results demonstrate that PGs can potentially act as vectors for the spread of *C. burnetii.* In some birds, *C. burnetii* was detectable in the serum, indicating that gulls can experience bacteraemia. It appears that different feeding strategies in the same species within the same ecosystem can have profound effects on the prevalence of pathogens. Further studies are required to better understand the epidemiology and potential risks of this organism.

## 1. Introduction

*Coxiella burnetii* is an intracellular zoonotic bacterial pathogen that is capable of causing a variety of symptoms in humans, ranging from mild flu-like symptoms through to cardiac disease and even fatalities [1,2]. It is a known cause of reproductive failure in wildlife species [3] and in recent years has been associated with declining populations of northern fur seals (*Callorhinus ursinus*) and Steller sea lions (*Eumetopias jubatus*) [4]. It was first described in marine mammals in the southern hemisphere, in 2022, in Australian fur seals (AusFS, *Arctocephalus pusillus doriferus*) breeding on Kanowna Island (KI) in the Bass Strait, southeastern Australia [5]. Australian fur seals have recently had decreased pup production, more obviously in their largest breeding colony at Seal Rocks [6]. A post-pupping environmental DNA prevalence of over 90% for *C. burnetii* has been detected in this breeding colony [7].

Birds are historically known to carry a variety of potentially zoonotic pathogens [8], yet wild birds not impacting domestic species have received little scientific attention as vectors of disease. Their role in the epidemiology of many important pathogens may thus be overlooked [9]. Gulls have nonetheless been the focus of a small number of zoonotic pathogen studies, as they are considered species likely to come into contact with human pathogenic enterobacterial organisms such as *Salmonella, Escherichia coli* and *Campylobacter sp.* [9]. It is apparent that they can disseminate antimicrobial-resistant *E. coli* over long distances [10]. Piscivorous birds and waterbirds such as gulls, that scavenge or feed on small invertebrates have recently been implicated as vectors of *Vibrio cholerae*, a deadly bacterial disease that can cause life-threatening diarrhea in human beings [11]. Historically, gulls have not been considered a significant vector of *C. burnetii*. However, they have recently been suspected as vectors of terrestrial genotypes of *C. burnetii* in colonies of northern fur seals [12].

The Pacific gull (PG, *Larus pacificus*) is the largest larid in Australia, with the biggest overall bill size and depth [13]. This adaptation allows PGs to consume large vertebrate prey such as small petrels (Procellariiformes) [13] but also allows them to scavenge and depredate thick-skinned chondrichthyans [14]. It has been observed that they consume AusFS placentas during the pupping season [5] and other gull species have also been observed as scavengers in northern fur seal breeding colonies [12].

**Figure 1:**
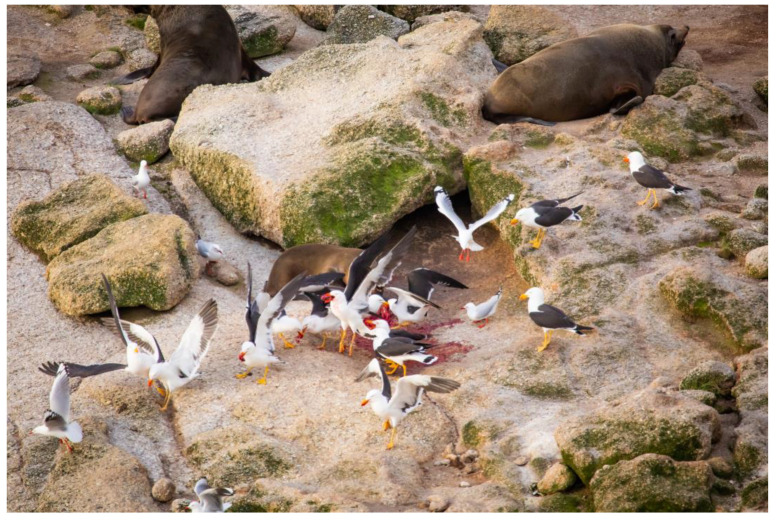
Pacific gulls scavenging an Australian fur seal placenta post-parturition.

The aim of this study was to determine if PGs could act as vectors of *C. burnetii* within the marine environment. Over the last decade, KI has been the focus of extensive AusFS research during the breeding season by some of the authors who have frequently observed that PGs scavenge freshly produced AusFS placental material (Figure 1). It was hypothesized, therefore, that they would acquire and act as vectors for this pathogen. Additionally, it was hypothesized that PGs breeding on Seal Island (SI), a nearby island where (despite the name) there is no AusFS breeding colony, would not have detectable *C. burnetii*. Considering that the role of birds in the epidemiology of *C. burnetii* is poorly understood [15], it is important to have a better grasp of the ecological role of the PG as a potential vector for spreading *C. burnetii* in marine mammal populations. Whether this pathogen could be a disease risk to the gulls themselves or pose a risk of being spread as a zoonotic infection are important considerations not investigated in this study.

## 2. Materials and Methods

### 2.1. Sampling

**Figure 2:**
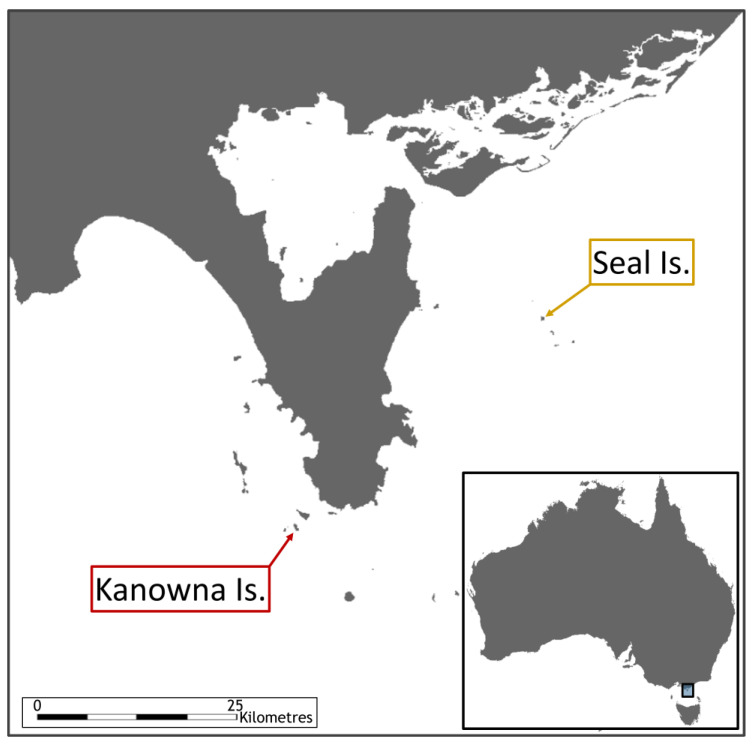
Map indicating the location of both sampling sites.

Adult PGs were captured during the AusFS breeding season, which coincides with incubation and early chick rearing for this bird species. Sampling occurred on KI (39°15′ S, 146°30′ E) and SI (38°92′ S, 146°66′ E) in Bass Strait (Figure 2). Kanowna Island is the third largest breeding colony for AusFS, with an approximate interannual pup production of 2200–4630 [16]. In contrast, SI has no AusFS or other species of pinniped that breed on the island. A total of 34 adult breeding individuals were sampled with 17 from each island. Birds were caught with a telescopic pole and noose system [17]. Oral and cloacal swabs were collected under manual restraint with a dry cotton swab and stored at −18 °C. Oral swabs were taken by focusing on swabbing all the oral cavity with special attention to the grooves along the hard palate and under the base of the tongue, where ingested material tended to be present. Cloacal swabs were gently inserted into the cloaca with a rolling action. Blood was collected (0.5–1.5 mL) from the medial metatarsal vein, allowed to stand for 30 min and centrifuged at 25× *g* for 15 min to separate the serum. Serum was stored at −18 °C. Each bird was identified with a metal leg band issued by the ABBBS (Australian Bird and Bat Banding Scheme). Handling and sampling were concluded in 10 min.

### 2.2. DNA Extraction

The swabs were incubated at 60 °C for 20 min in 400 µL of phosphate buffered saline. Genomic DNA was extracted and purified from the swabs using a HiYield Genomic DNA Mini Kit (Real Biotech Corporation, Banqiao City, Taiwan) as per the manufacturer details. The same kit was used to extract genomic DNA from the serum samples. All extracted DNA was assessed for quality and purity of extracted nucleic acids using a Nanodrop ND-1000 spectrophotometer (Thermo Fisher Scientific, Waltman, MA, USA).

### 2.3. Quantitative Polymerase Chain Reaction

Samples were tested with three different quantitative polymerase chain reaction (qPCR) techniques. The targets were the com1 and htpAB genes and the IS1111 insertion. The same qPCR techniques were used as employed by researchers in the initial detection of *C. burnetii* in AusFS [5], apart from the addition of the IS1111 insertion. The IS1111 marker is presumed to be absent from *C. burnetti* in AusFS [7,12]. The IS1111 markers were based on previously described techniques [18].

A volume of 5 µL of extracted DNA was used for each sample in a total cycle volume of 25 µL. All samples were run together with a no template control of 5 µL of nuclease free water (Gibco, Mulgrave, VIC, Australia). The positive control was 5 µL Nine Mile Phase II, Clone 4 (RSA439), which was obtained after repeated passage in Vero cells. Amplifications were performed in a magnetic induction PCR cycler (Mic), using Mic PCR software, version 2.8.13. Green fluorescence (uracil DNA glycosylase) was detected at 50 °C for 3 min. Activation was achieved at 95 °C for 5 min with a platinum Taq DNA polymerase. All three primers went through 40 cycles of 95 °C for 20 s to achieve denaturation, and 60 °C for 40 s to allow for annealing and elongation.

All three markers are semi-quantified using real-time TaqMan (qPCR) assays using a proprietary Invitrogen Platinum Quantitative PCR SuperMix-UDG (Thermo Fisher Scientific, Waltman, MA, United States). Details of all primers and assays used for placental tissue and eDNA *C. burnetii* detection are summarized in Table 1.

## 3. Results

The conversion threshold (Ct) values were determined as a range with a mean for each sample type. The Ct for the com1 oral swabs from KI had a range of 18.72–37.38 with a mean of 31.56 and the htpAB oral swabs had a range of 19.71–38.5 with a mean of 31.65. Only one oral swab from SI was amplified and solely on the htpAB with a Ct of 37.99. The Ct values for both the com1 and htpAB were higher for the cloacal swabs from KI compared to the oral swabs. The com1 values ranged from 28.18 to 38.25 with a mean of 34.05 and the htpAB values ranged from 28.73 to 38.5 with a mean of 34. Comparatively, the range for both com1 and htpAB were higher for SI. The com1 ranged from 31.39 to 37.11 with a mean of 35.06 and the htpAB had a range of 31.29–36.2 with a mean of 33.62. Overall, amplification was low on serum samples and the only positive samples were obtained from KI. The com1 ranged from 34.27 to 37.55 and the htpAB ranged from 37.22 to 37.89. Means were not calculated for the serum samples, or the SI swabs due to low numbers of the samples showing amplification. Ct values above 40 were considered negative.

**Figure 3:**
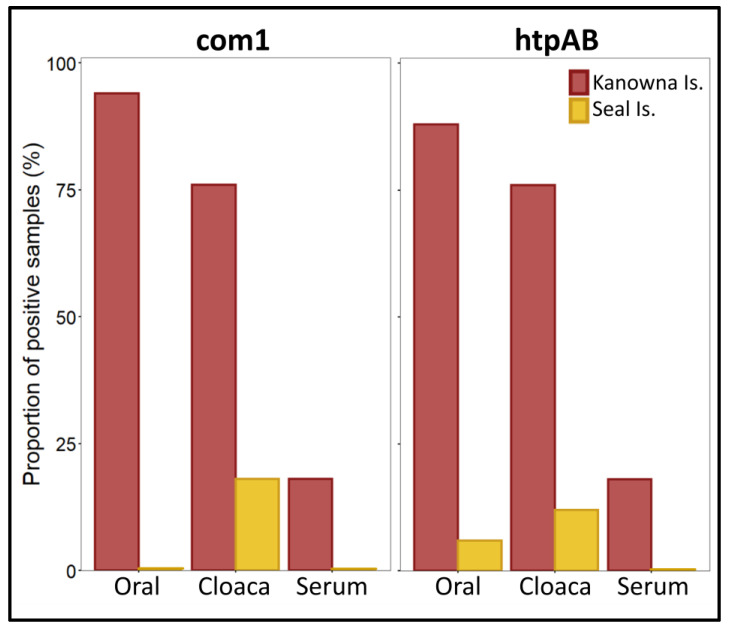
Histogram of com1 and htpAB for all three sample types from both Kanowna Island and Seal Island.

Sixteen out of 17 (94.1%) of the oral swabs from KI tested positive on the com1 and 15/17 (88.2%) on the htpAB targets (Table 2; Figure 3). Oral swabs did not test positive on the com1 (0%) and only 1/17 (5.9%) on the htpAB targets for the SI colony (Table 2; Figure 3). The cloacal swabs from KI tested positive on both com1 and htpAB in 76.5% (n = 13/17) samples, whereas the SI colony had positive results of 17.7% (n = 3/17) and 11.8% (n = 2/17), respectively (Table 2; Figure 3). Only 17.7% (n = 3/17) of serum samples from KI were positive for either the com1 or htpAB targets, whereas none were positive from the SI colony (Table 2; Figure 3). No samples from either colony tested positive on the IS1111 target. Table 2 summarizes the qPCR results across all three target sequences and all three sample types from both KI and SI.

## 4. Discussion and Conclusions

The results of the present study suggest that PGs play a role in the epidemiology of *C. burnetii* in AusFS breeding colonies. The high prevalence of oral and cloacal swabs of PGs sampled on KI in the present study supports the hypothesis that most gulls feeding on placentas at this location are exposed to *C. burnetii.* Kanowna Island is the third largest breeding colony for AuFS, having approximately 15,000 animals present on the island [6] and more than 2200 pups born annually [16]. A prevalence of up to 56.7% for *C. burnetii* in AusFS placentas has been reported from KI during the peak of pupping [7]. Therefore, a high density of potentially infected placental material is produced over a very concentrated pupping season.

From the results presented here, it can be deduced that PGs might be able to serve as mechanical vectors for *C. burnetii.* Both their ability to mechanically transmit and amplify infection requires further studies. There are no reports of PGs being negatively affected by *C. burnetii* or whether pathology develops within the gulls themselves. Migratory birds receive considerably more attention as vectors of emerging infectious diseases and especially those that are considered zoonotic [8]. Although the PGs in the Bass Strait are not classified traditionally as migratory birds, given their likely role as mechanical vectors, it would be pertinent to determine if there is any seasonal short distance shift or variation in their foraging that could allow them to spread *C. burnetii*.

It is unknown if the *C. burnetii* detected in the cloacal swabs of the gulls examined here would be viable. However, it is most likely that the small cell variant, which is highly resistant to environmental degradation, is being excreted in faeces [2]. With 47.1% of all cloacal swabs being positive for *C. burnetii* DNA, there is a substantial probability that PG could disseminate the organism throughout the marine and coastal environments. The detectable presence of *C. burnetii* in the faeces of birds in other studies has been linked to the spread of *C. burnetii* into the environment through avian vectors [15]. Faecal shedding could further be prolonged in birds that are subclinically infected. Currently, it is not known whether subclinical infections in birds occur, but this has been shown in cattle where infection persists for months with shedding of organisms in multiple body fluids and secretions [19]. If gulls feeding on infected placenta can become subclinically infected, it would be very important to understand how and where they are able to transmit the pathogen. At present, it is unknown if gulls could develop an undetectable carrier state with a quiescent infection.

The number of positive samples from gulls at both colonies was much lower for serum than cloaca or oral cavity. This likely due to the brief period in which *C. burnetii* is detectable in serum before an immune response is raised [18]. In a study across multiple migratory and resident bird species in the Baltic region, it was found that only 1.4% of blood samples tested positive for the presence of *C. burnetii* using the com1 target, some of which were black-headed gulls (*Larus ridibundus*) [20]. The study concluded that migratory birds could transmit infection to ticks but did not venture into whether birds without ticks could act as vectors of *C. burnetii* [20]. It may be that many more birds would actually have detectable *C. burnetii* DNA if tested soon after infection [21].

It appears that PGs feeding within AusFS colonies during the pupping season are much more likely to be exposed and potentially transmit *C. burnetii* than gulls with a non-placenta-based diet over the same period. These two PG colonies are less than 40 km apart (Figure 2). It is apparent that potentially different feeding strategies in the two PG colonies sampled, cohabiting in the same Bass Strait ecosystem, can have profound effects on the prevalence of a pathogen such as *C. burnetii*. It has been shown that PGs are able to travel extensive distances between their breeding colonies and foraging grounds, allowing them to act as a vector of anthropogenic debris [22]. While the exact foraging strategy of both gull populations is currently data deficient, this study highlights how variations in the availability of proximate resources may influence the spread of pathogens.

The complete lack of IS1111 amplification in all samples from both PG colonies further supports the hypothesis that *C. burnetii* in AusFS is potentially not related to terrestrial Australian genotypes of the organism but rather closer in relation to marine mammal strains detected in the northern hemisphere [5,12]. In two extensive studies of marine mammals, it has been found that placental tissue fails to show or shows poor amplification of IS1111 [7,12]. As this is a multicopy with high sensitivity [18], it has been proposed as a potential tool to distinguish between terrestrial and marine mammal-derived strains [7,12].

It would be important to determine if PGs feeding on placental material on KI remain on the island during the AusFS breeding period or have flight paths to other distant islands or the mainland, where they could be spreading the pathogen. This is important for understanding the potential spread and how it might impact other susceptible species such as livestock, humans, and other marine mammals.

## Figures and Tables

**Table 1:** Primers and probes used in qPCR assays for the detection of Coxiella burnetii DNA in oral swabs, cloacal swabs and serum from Pacific gulls (Larus pacificus) from Kanowna Island and Seal Island. All primers and probes were synthesized by Integrated DNA technologies (IDT).

Assay	Primer/Probe	Sequence(5’–3’)	Final Concentration (NM)	Amplicon Size (BP)
com1	com1_F	AAAACCTCCGCGTTGTCTTCA	400	76
	com1_R	GCTAATGATACTTTGGCAGCGTATTG	400	
	com1_P	FAM ^a^-AGAACTGCCCATTTTTGGCGGCCA-BHQ1 ^b^	200	
htpAB	htpAB_F	GTGGCTTCGCGTACATCAGA	400	114
	htpAB_R	CATGGGGTTCATTCCAGCA	400	
	htpAB_P	FAM-AGCCAGTACGGTCGCTGTTGTGGT-BHQ1	200	
IS1111	IS*1111*NL_F	AAAACGGATAAAAAGAGTCTGTGGTT	300	70
	IS*1111*NL_R	CCACACAAGCGCGATTCAT	300	
	IS*1111*NL_P	Quasar 670 ^c^-AAAGCACTCATTGAGCGCCGCG-BHQ2 ^d^	150	

^a^ 6-Carboxyfluorescein. ^b^ Black Hole Quencher-1. ^c^ Quasar 670 carboxylic acid. ^d^ Black Hole Quencher.

**Table 2:** Prevalence of *Coxiella burnetii* from oral swabs, cloacal swabs and serum collected from Pacific gulls (*Larus pacificus*) breeding on Kanowna Island and Seal Island based on qPCR assays of com1, htpAB and IS1111. n = Sample size.

	n	Com1	htpAB	IS1111	Both Com1 and htpAB
Kanowna Island					
Oral swab	17	16 (94.1%)	15 (88.2%)	0 (0%)	15 (88.2%)
Cloacal swab	17	13 (76.5%)	13 (76.5%)	0 (0%)	7 (41.2%)
Serum	17	3 (17.7%)	3 (17.7%)	0 (0%)	2 (11.8%)
Seal Island					
Oral swab	17	0 (0%)	1 (5.9%)	0 (0%)	0 (0%)
Cloacal swab	17	3 (17.7%)	2 (11.8%)	0 (0%)	2 (11.8%)
Serum	11	0 (0%)	0 (0%)	0 (0%)	0 (0%)

## Data Availability

Data is available on request.
